# Bridging the gaps in newborn screening programmes: Challenges and opportunities to detect haemoglobinopathies in Africa

**DOI:** 10.4102/ajlm.v12i1.2225

**Published:** 2023-12-14

**Authors:** Seth Twum, Kwadwo Fosu, Robin A. Felder, Kwabena A.N. Sarpong

**Affiliations:** 1 West African Centre for Cell Biology of Infectious Pathogens, Accra, Ghana; 2 Department of Biochemistry, Cell and Molecular Biology, University of Ghana, Accra, Ghana; 3 Department of Pathology, The University of Virginia, Charlottesville, Virginia, United States

**Keywords:** haemoglobinopathies, sickle cell disease, newborn screening, Africa, thalassaemia

## Abstract

**Background:**

Haemoglobinopathies, including sickle cell disease and β-thalassaemia, are monogenic disorders with a relatively higher prevalence among malaria-endemic areas in Africa. Despite this prevalence, most African countries lack the necessary resources for diagnosing and managing these debilitating conditions.

**Aim:**

This study provides a critical review of newborn screening for detecting haemoglobinopathies in Africa, highlighting challenges and proposing strategies for improved diagnosis and management.

**Methods:**

A literature search on haemoglobinopathies in Africa was conducted in PubMed, Google Scholar and ScienceDirect, using specific keywords and Boolean operators, including articles published from January 1981 to December 2022.

**Results:**

The data show that sickle cell disease is prevalent among populations in Central and West Africa; however, β-thalassaemia is prevalent among people in the northern parts of Africa. Newborn screening pilot initiatives for haemoglobinopathies were being implemented in Angola, Nigeria, Ghana, the Democratic Republic of Congo and the Republic of Benin. The cost of testing, lack of sufficient and accessible medical records, and inadequacy in healthcare infrastructure pose significant challenges in bridging the gaps in newborn screening. Furthermore, the stigmatisation and lack of awareness of haemoglobinopathies and access to newborn screening programmes pose additional challenges.

**Conclusion:**

This review highlights the challenges associated with haemoglobinopathy testing, effective strategies for mitigating these challenges, and future perspectives for expanding efforts toward detecting and managing these disorders across Africa. Providing affordable diagnostic tools, mobile clinics, government subsidies, education campaigns, and the implementation of electronic medical records systems could help bridge the gaps in newborn screening in Africa.

**What this study adds:**

The study presents a comprehensive view of newborn screening of haemoglobinopathies in Africa, provides a detailed outline of the challenges faced by newborn screening for haemoglobinopathies in Africa, and offers strategies for better diagnosis and care.

## Introduction

Haemoglobinopathies such as sickle cell disease (SCD) and β-thalassaemia are global health monogenic disorders with a significantly higher prevalence among people in malaria-endemic areas owing to natural selection.^1^ About 70% of the recorded global haemoglobinopathy cases are in Africa. Additionally, Africa has the highest under-five mortality for haemoglobin disorders among affected children.^2^ These haemoglobinopathies are inherited autosomal recessive haemoglobin abnormalities. Clinically significant haemoglobinopathies include α- and β-thalassemia, SCD, haemoglobin E and haemoglobin C.^3^ In sub-Saharan African populations, SCD is prevalent (10.68%) and is caused by abnormal haemoglobin resulting from the substitution of valine for glutamic acid at position six of the β-globin chain.^4^ At low oxygen tension, sickle blood (HbS) polymerises easily, affecting red blood cell structure, function and life span.^5^ Heterozygous HbS individuals are generally asymptomatic. In contrast, homozygous HbS individuals show clinical symptoms of SCD.^6^

Since the development of HbS newborn screening (NBS) programmes, it has become easier to identify babies with haemoglobin abnormalities at birth or in the first few days of life, before they exhibit clinical signs.^7^ According to the World Health Organization, haemoglobinopathy screening aims to identify carriers to estimate the risk of birthing children with haemoglobinopathies, followed by genetic counselling and offering options to avoid such births. In high-income countries, patients are engaged in programmes that provide strategies for complete care, resulting in better outcomes than in poorly resourced countries where comprehensive NBS programmes are not implemented. In 2019, Africa recorded 38 403 SCD deaths, accounting for, approximately, a 26% increase in SCD mortality in children compared to 2000.^8^ Without appropriate interventions, it is estimated that 90% of people suffering from SCD in Africa might not survive beyond 18 years of age.^9,10,11^ Early NBS for SCD allows for adopting a complete care strategy that includes preventative therapy, parental education, and the start of a tracking and follow-up programme for diagnosed individuals.^12^ Sickle cell diseases are among the most important illnesses because of their prevalence and effects in sub-Saharan Africa, making this group of disorders the most vital to test for NBS in a significant portion of the continent,^13^ yet resources have not been made available by governments in sub-Saharan Africa and other high-prevalence countries.^14^

Haemoglobinopathy diagnosis is achieved using the sickling test as a screening modality, followed by haemoglobin electrophoresis. Isoelectric focusing and high-performance liquid chromatography are considered gold standards for sickle cell diagnosis as they differentiate between normal sickle cell trait and SCD.^15^ Other diagnostic tests, such as multiplex ligation probe amplification and gap polymerase chain reaction, are also offered at selected facilities. The development of point-of-care testing for SCD^15,16^ is very beneficial, especially in locations with limited capabilities for central laboratory testing or where the transfer of blood samples to a centralised laboratory is not practical or convenient. The prevalence of haemoglobinopathy, genetic variability and cost efficiency are the primary variables influencing the choice of a specific haemoglobinopathy screening technique. Mass screening programmes targeting people at various phases of life can be implemented where prevalence is high.^17^ However, testing for haemoglobin disorders in Africa is associated with numerous challenges that require attention and practical solutions. This article examines interventions related to diagnosing haemoglobinopathies in Africa, including various diagnostic methodologies. It highlights valuable lessons from past experiences, focusing on the challenges associated with haemoglobinopathy testing and effective strategies for mitigating them. Additionally, the article provides future perspectives for expanding efforts toward detecting and managing these disorders across Africa.

## Methods

### Literature search and data sources

**FIGURE 1:**
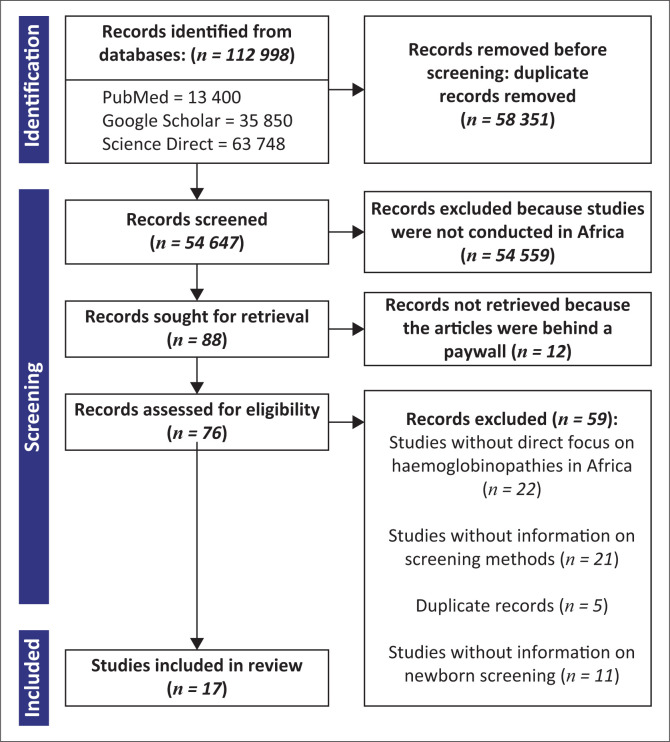
Flow chart of manuscripts reporting on haemoglobinopathies in Africa. Electronic literature search between 1981 and 2022 was performed using specific keywords, inclusion and exclusion criteria to identify manuscripts that have published on haemoglobinopathies in Africa.

The literature search was carried out through PubMed^®^ (https://pubmed.ncbi.nlm.nih.gov/), Google Scholar (https://scholar.google.com/), and ScienceDirect (https://www.sciencedirect.com/), employing a combination of Boolean operators (and, or) and predetermined keywords. Our search terms were ‘hemoglobinopathies and Africa or blood disorders and Africa’, ‘sickle cell disease and Africa’, and ‘thalassemia disease and Africa’. To ensure the relevance of our findings, we restricted the search to publications between January 1981 and December 2022. The titles of the articles were selected based on the following eligibility criteria: (1) the study location must be within Africa, and (2) the study must have reported the detection of sickle cell or thalassaemia disease or both. For an article to be considered, it must have reported the number of newborns screened, the number of haemoglobinopathies detected, and the testing method employed to identify the specific haemoglobinopathy. Studies without a direct focus on haemoglobinopathies in Africa and studies that did not provide information on the method used for screening were excluded (Figure 1). Research articles and systematic reviews with metadata that suited the inclusion criteria were retrieved. In addition, we conducted supplementary validation by thoroughly reviewing the references cited in the eligible articles.

### Data extraction, analysis, and visualisation

Data were extracted into a Microsoft Office Excel 2016 (Microsoft Corporation, Redmond, Washington, United States) spreadsheet. For each eligible study, data extracted included study location or geographical zone, publication year, screening period, newborns screened, haemoglobinopathies tested, and the testing method used. The percentage prevalence of sickle cell and thalassaemia diseases reported for each study was calculated by dividing the number of haemoglobinopathies detected by the total number of newborns screened for haemoglobinopathies, multiplied by 100. Packages found in R-studio (Posit, Public-Benefit Corporation, Vienna, Austria), such as ggplot2 and tidyverse, were used to generate the geographical zones with colour codes, using the calculated prevalence.

## Results

### Intervention: Haemoglobinopathies tested in Africa

According to the World Health Organization, in 2010, defective haemoglobin genes affected around 5% of the world’s population; more than 300 000 infants are born with clinically severe haemoglobin abnormalities yearly. Research shows these figures will increase from 305 800 in 2010 to 404 200 in 2050.^11^ Although about 80% of affected children are born in underdeveloped countries, 83% are born with SCD, with the remainder having thalassaemia syndromes.^16^ About 50% to 80% of children with SCD and more than 50 000 children with major thalassaemia die in low- and middle-income countries.^11^ Fortunately, NBS with comprehensive care for SCD has been found to reduce early child mortality.^17^ As of 2023, no African country had succeeded in making SCD a universal national health intervention.^18,19,20^ However, pilot screening programmes have been conducted by the member countries of the Consortium on Newborn Screening in Africa, such as Kenya, Ghana, Liberia, Nigeria, Tanzania, Zambia and Uganda, and by other non-consortium members, such as Benin, Burkina Faso, Democratic Republic of Congo, Gabon and Mali.

**FIGURE 2:**
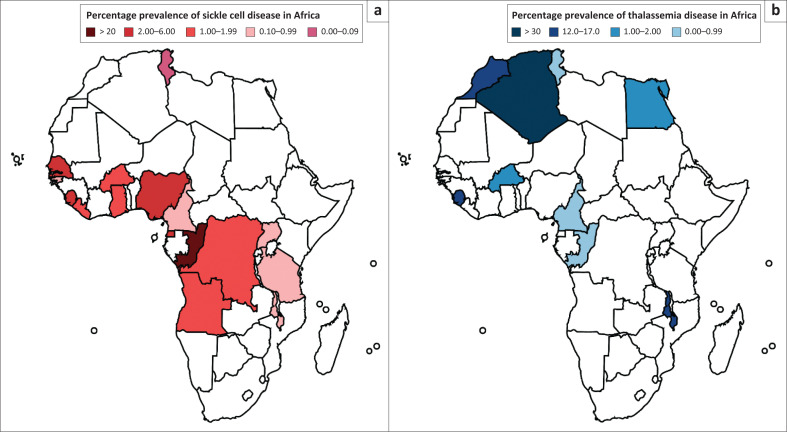
Prevalence of haemoglobinopathies in Africa (a) sickle cell disease (b) α- and β-thalassaemia, 1981–2022.

The data analysis from NBS programmes in Africa shows that SCD is prevalent among populations in Central and West Africa (Figure 2a). More SCD prevalence exists in individuals residing in the Democratic Republic of Congo. Conversely, β-thalassemia is prevalent among people in the northern parts of Africa (Figure 2b), with Algeria and Morocco having the highest prevalence.^21^ In Algeria and Morocco, ~75% are Caucasians of Arabic origin, while the Congolese are multi-ethnic, with a significant percentage of black people. It is well documented that Arab countries, such as Morocco, have consanguinity of up to 30% and contribute significantly to the increased prevalence of inherited metabolic errors.^22^ Conversely, while SCD affects approximately 1 in 365 live births among Africans, the sickle cell trait is found in about 1 in 13 Africans.^23^ Apart from Tunisia, where the prevalence of SCD is 9 in 10 000, the incidence of SCD is high in the western and central parts of Africa.

Although the lack of testing and the limited literature have contributed to the scarcity of data on haemoglobinopathy testing in Africa, some countries have been making massive efforts to pilot robust NBS programmes to identify a selected number of these conditions. Our literature search made it increasingly apparent that obtaining data for haemoglobinopathy testing and disease prevalence for more than 50% of African countries became difficult. From 2014 to 2019, Uganda screened more than 220 000 newborns in Africa (Table 1) and is one of the African countries involved in mass screening through pilot programmes. In addition, other countries such as Ghana and Angola participate in mass testing via pilot programmes (Table 1). Isoelectric focusing has been Africa’s most widely used diagnostic haemoglobinopathy technique due to its higher resolution, low cost, and effortless performance.^24^

**TABLE 1:** Haemoglobinopathies tested in Africa and the test method obtained from electronic literature search between 1981 and 2022.

Country	Year(s)	Number of newborns screened	Haemoglobinopathy tested				Method(s)	Ref.
			HbS	Thala (α/β)	% HbS tested	% Thala tested		
Algeria	1981	293	n/a	Y	0	34.5	CAE	^25^
Republic of Congo	2019–2020	2897	Y	Y	20.6	0.035	HPLC	^26^
Liberia	2010–2012	3986	Y	N	1.2	0	IEF	^18^
Angola	2011–2013	36 453	Y	Y*	1.51	n/a	IEF, CE	^27^
DR Congo	2018	310	Y	N	1.9	0	IEF, CE	^28^
Tanzania	2015–2016	3981	Y	N	0.8	0	IEF, HPLC	^29^
Nigeria	2008	644	Y	N	3.0	0	IEF	^18^
Ghana	1991–2020	523 159	Y	N	1.9	0	IEF	^18^
Benin	1993–1996	1189	n/a	N	0	0	IEF	^30^
Botswana	n/a	-	-	-	-	-	-	-
Burkina Faso	2000–2004	2341	Y	Y (α)	1.8	1.71	IEF, CE and HPLC	^31^
Burundi	n/a	-	-	-	-	-	-	-
Cameroon	2016	703	Y	Y (β^+^)	0.71	0.57	HPLC	^32^
Cape Verde	n/a	-	-	-	-	-	-	-
Central African Republic	n/a	-	-	-	-	-	-	-
Chad	n/a	-	-	-	--	-	-	-
Comoros	n/a	-	-	-	-	-	-	-
Djibouti	n/a	-	-	-	-	-	-	-
Egypt	2018	303	n/a	Y (β)	0	1	HPLC	^33^
Equatorial Guinea	2017	328	Y	N	2.9	0	IEF	^34^
Eritrea	n/a	-	-	-	-	-	-	-
Eswatini	n/a	-	-	-	-	-	-	-
Ethiopia	n/a	-	-	-	-	-	-	-
Gabon	n/a	-	-	-	-	-	-	-
Gambia	n/a	-	-	-	-	-	-	-
Guinea	n/a	-	-	-	-	-	-	-
Guinea-Bissau	2012	848	Y	n/a	0.94	0	HPLC	^35^
Ivory Coast	n/a	-	-	-	-	-	-	-
Kenya	n/a	-	-	-	-	-	-	-
Lesotho	n/a	-	-	-	-	-	-	-
Libya	n/a	-	-	-	-	-	-	-
Madagascar	n/a	-	-	-	-	-	-	-
Malawi	2018	10 529	Y	Y (α)	0.1	12.62	IEF	^36^
Mali	n/a	-	-	-	-	-	-	-
Mauritania	n/a	-	-	-	-	-	-	-
Mauritius	n/a	-	-	-	-	-	-	-
Morocco	2015–2016	1658	N	Y (α)	0	16	Gap-PCR, CE, MLPA	^37^
Mozambique	n/a	-	-	-	-	-	-	-
Namibia	n/a	-	-	-	-	-	-	-
Niger	n/a	-	-	-	-	-	-	-
Rwanda	n/a	-	-	-	-	-	-	-
Sao Tome and Principe	n/a	-	-	-	-	-	-	-
Senegal	2003	478	Y	N	2.1	0	IEF	^18^
Seychelles	n/a	-	-	-	-	-	-	-
Sierra Leone	2013	388	Y	Y (β)	5.4	16.5	Agarose gel	^38^
Somalia	n/a	-	-	-	-	-	-	-
South Africa	n/a	-	-	-	-	-	-	-
South Sudan	n/a	-	-	-	-	-	-	-
Sudan	n/a	-	-	-	-	-	-	-
Togo	n/a	-	-	-	-	-	-	-
Tunisia	2010	9148	Y	Y (β)	0.01	0.01	IEF, HPLC	^39^
Uganda	2014–2019	220 827	Y	N	0.92	0	IEF	^40^
Zambia	n/a	-	-	-	-	-	-	-
Zimbabwe	n/a	-	-	-	-	-	-	-

HbS, sickle cell haemoglobin; Thala, thalassaemia; Ref., reference; MLPA, Multiplex Ligation Probe Amplification; CE, capillary electrophoresis; CAE, cellulose acetate electrophoresis; IEF, isoelectric focusing; HPLC, high-performance liquid chromatography; Gap-PCR, gap polymerase chain reaction; n/a, no data available; Y, present; N, absent; Y*, not quantified; α, alpha-thalassemia; β, beta-thalassemia; DR Congo, Democratic Republic of Congo.

A study was conducted in the northern part of Algeria and α-thalassemia was prevalent.^25^ In Morocco, a study was conducted in three of the northern provinces, and α-thalassemia was also prevalent.^38^ For the Republic of Congo, a study was conducted in 12 departments in the entire national territory of Congo, and SCD was prevalent.^26^ In Malawi, a study was conducted in the central region of Malawi, and α-thalassemia was prevalent.^37^ In Sierra Leone, a study was conducted on selected participants from the rural and urban areas, and β-thalassaemia was prevalent.^39^

## Discussion

### Challenges and mitigation strategies for haemoglobinopathy testing

Testing for haemoglobinopathies is essential for early diagnosis and appropriate treatment. However, several challenges and limitations are associated with haemoglobinopathy testing in Africa. Here, we discuss the critical challenges with haemoglobinopathy testing in Africa and strategies to mitigate these challenges.

#### Point-of-care technologies

Many African countries lack the necessary resources, equipment and personnel with advanced training to perform testing and accurately interpret test results. Most African countries may not have financial or technical support to establish and maintain a robust laboratory infrastructure for haemoglobinopathy testing.^41^ A potential solution to this challenge is using affordable diagnostic tools, such as rapid diagnostic tests, that are simple and easy to use.^42^ Sickle cell analysis (BioMedomics Incorporated, Morrisville, North Carolina, United States),^43^ lateral flow immunoassays,^44^ and HemoType SC (Silver Lake Research Corporation, Azusa, California, United States)^45,46^ are affordable, user-friendly, quick diagnostic tests that can successfully be used in resource-limited settings. Investing in affordable point-of-care diagnostic tools can help improve the diagnosis and management of haemoglobinopathies in Africa.

#### Mobile clinics

In Africa, a significant portion of the population resides in areas that are geographically distant from testing facilities,^47^ making it difficult to access testing centres or clinics that diagnose haemoglobinopathies. As a result, they may not have access to essential health services, including diagnostic testing, which can lead to increased health risks and delays in treatment. Mobile clinics could solve this challenge by directly bringing healthcare services to underserved communities, including remote areas. Using mobile clinics and community-trained laboratory personnel, healthcare providers can improve access to essential health services for people living in remote areas. Furthermore, mobile clinics can meet the specific healthcare needs of different communities, and they can offer a wide range of services beyond diagnostic testing, including vaccination, health education, and counselling.

Additionally, Africans’ rapid internal and external migration has made it difficult to estimate and control newborn haemoglobinopathies accurately. Rapid migration changes epidemiology since people from high-prevalence areas move to low-prevalence areas.^45^ Migrations without universal electronic health records management make it difficult to follow up with parents with these haemoglobinopathies and their newborns. Specifically, there is a high-prevalence of HbS in Africa and haemoglobin C in parts of West Africa.^46^ Without proper measures, heterozygous haemoglobin C may spread due to migration if SCD is not controlled, increasing the likelihood of more HbS cases in Africa. Through an influx of migrants from West and Central Africa, internal migration in Africa has caused SCD, a previously rare disease, to be introduced in South Africa.^4^

#### Government subsidies

The cost of testing and treating various medical conditions in Africa can be prohibitively expensive for many low-income patients.^48^ This financial burden can prevent people from seeking the care they need, leading to delayed diagnoses and life-threatening consequences. Governments in Africa have a role to play in addressing this problem. By creating programmes subsidising the cost of testing and treatment for low-income individuals, they can ensure everyone has access to quality healthcare regardless of their ability to pay. These programmes could take various forms, including public health insurance schemes, direct subsidies for medical expenses, or partnerships with private healthcare providers to offer discounted rates for low-income patients. In addition to making care more affordable, these initiatives could also help to improve the overall quality and availability of healthcare services across the continent. By prioritising access to healthcare for all, African governments can help address SCD, one of the most pressing challenges facing the continent today.

#### Awareness campaigns

Despite the prevalence of haemoglobinopathies, many Africans are unaware of these disorders and their potential impact on health.^48^ One consequence of this lack of awareness is a low demand for testing, which can result in delayed diagnosis and treatment. Also, access to newborn babies within days of birth or non-newborns is challenging in Africa because women living in rural areas underutilise formal and evidence-based maternal health delivery services.^49,50^ Awareness campaigns through various traditional and digital media platforms can help address these issues. Traditional media platforms such as television, radio, and print media can reach a broad audience, especially in rural areas with limited access to the Internet. These campaigns can educate people about the symptoms of haemoglobinopathies, the importance of testing, and the available treatment options. In addition, digital media platforms, such as social networks and mobile applications, can also be used to raise awareness.^19^ Creating awareness of haemoglobinopathies is crucial for ensuring that people affected by these disorders receive adequate and timely medical care.

#### Targeted educational programmes

Some African communities stigmatise individuals with haemoglobinopathies due to cultural beliefs and misconceptions.^51^ This stigmatisation often arises from a lack of understanding of the genetic and biological basis of haemoglobinopathies, which can lead to harmful and discriminatory attitudes toward individuals with the condition.^52^ Some communities view haemoglobinopathies as a result of witchcraft, curses, or other supernatural causes, leading to fear, ostracism and discrimination.^53^ Education campaigns can address these beliefs and reduce stigmatisation. By raising awareness of the genetic and biological causes of haemoglobinopathies and the impact of stigmatisation on individuals and communities, education campaigns can help dispel myths and misconceptions surrounding the condition.^54^ Education campaigns can also promote acceptance and support for individuals with haemoglobinopathies, encouraging communities to recognise and value the contributions of all members.^55^ By challenging stereotypes and promoting inclusion, education campaigns can help to create more inclusive and supportive communities that prioritise the health and well-being of all members, regardless of their health status.

#### Secure but accessible electronic medical record systems

Furthermore, in most African countries, medical records are not easily accessible, making it difficult to track the prevalence of haemoglobinopathies and monitor a patient health outcomes.^50^ Implementing electronic medical records systems is a potential way to overcome this challenge. These electronic medical records are digital versions of patients’ medical records that healthcare providers can access and update in real time. Electronic medical records can help healthcare providers collect and store patient health information, including diagnostic test results, medical histories, and treatment plans. Electronic medical records can help to improve the accuracy and completeness of patients’ medical records and facilitate communication between healthcare providers, which is particularly important for patients with chronic conditions, such as haemoglobinopathies. They can also help to improve patient outcomes by enabling healthcare providers to monitor patient’s health over time, tracking medication adherence, and identifying potential complications or drug interactions. Accessible electronic medical records can lead to earlier interventions and more personalised care, ultimately improving patients’ quality of life and reducing the burden of haemoglobinopathies on healthcare systems.

#### Standardised quality control material

Additionally, the quality of testing for haemoglobinopathies in Africa is a major concern due to poor quality assurance practices. Quality assurance is crucial in haemoglobinopathy testing to avoid misdiagnosis, which can have severe consequences for patients, including incorrect treatment and poor health outcomes. Unfortunately, many testing facilities in Africa lack quality assurance programmes or have inadequate ones, which can lead to errors in testing and misdiagnosis.^56^ Quality assurance programmes not only need specimens with known values to measure along with patient specimens but also involve regular checks of equipment, reagents, and personnel performance, as well as ongoing training and education of staff on quality assurance practices. Quality assurance includes standardisation and harmonisation in testing protocols between laboratories and between countries.^19^

### Perspectives for expanding the determination of haemoglobinopathies in Africa

In Africa, interventions to control haemoglobinopathies focus on using primary healthcare and health promotion strategies to ensure policy formulation and implementation, legislation and regulation, and improving primary and secondary prevention measures.

One of the main strategies is to partner with local health organisations.^19^ Partnerships with local health organisations involve working with hospitals, clinics, community health centres, and other organisations to develop educational materials and outreach programmes to raise awareness about haemoglobinopathies and their impact on health.

National programmes for haemoglobinopathies, especially SCD and thalassaemia disorders, must be established and strengthened to prevent and control non-communicable diseases and should work together with national programmes for maternal and child health.^57^ Key focus areas include advocacy, prevention and counselling, early detection and management, data collection and monitoring, research, community education, and collaboration. An integrated, multi-sectoral, and multi-disciplinary team of healthcare and social workers, teachers, parents, and concerned non-governmental organisations could address these areas. This team would focus on practical aspects of care delivery and programme implementation and monitoring.

Additionally, establishing genetic counselling and testing measures is essential.^58^ Through genetic counselling, carriers of haemoglobinopathies can learn the risks of passing the disease on to their children, and receive guidance on family planning and reproduction options; this can help prevent the transmission of haemoglobinopathies through informed decision-making by families. Widespread community participation and support are required to ensure individuals have access to these services and promote awareness and education about haemoglobinopathies.

Implementing community-based actions, such as surveillance and supervision, monitoring at all levels of operation, and periodic national review, will ensure that accurate and timely data is collected and used to guide decision-making.^57^ Community-based surveillance and supervision can involve training community health workers to identify and report cases of haemoglobinopathies and providing education and support to affected individuals and families. Monitoring at all operation levels, from the local to the national level, can help ensure that data are collected consistently and accurately and that appropriate data-backed interventions are implemented. Periodic national reviews can identify haemoglobinopathy prevalence and incidence trends and surveillance system gaps. These data can then inform policy making and day-to-day programme management decision-making, such as allocating resources for prevention and treatment programmes, developing guidelines for screening and diagnosis, and providing training and support for healthcare providers.

Promoting pilot programmes is another strategy for expanding haemoglobinopathy detection in Africa. Newborn screening programme pilot initiatives for haemoglobinopathies are being implemented in Angola,^27^ Nigeria,^59^ Ghana,^60^ the Democratic Republic of Congo,^26,61^ and the Republic of Benin.^30^ However, many African countries do not have universal national NBS programmes for SCD and other haemoglobinopathies.

### Summary

Haemoglobinopathies, such as SCD and β-thalassemia, are genetic disorders caused by abnormal haemoglobin affecting red blood cells. In Africa, several challenges limit the testing for these disorders, including a lack of resources, limited access to testing facilities, migration, financial constraints, lack of awareness, and stigma. To improve access to quality healthcare services and raise awareness of African haemoglobinopathies, we propose using affordable diagnostic tools, mobile clinics, government subsidies, education campaigns, and establishing records or registries. The future may see these proposed strategies implemented to improve healthcare services for African haemoglobinopathies.
